# The Medical Association of Georgia

**Published:** 1888-05

**Authors:** 


### THE MEDICAL ASSOCIATION OF GEORGIA.

*Editors Atlanta Medical and Surgical Journal:*

The Medical Association of Georgia convened in Rome for its 39th annual session on Wednesday, April 18th, 1888. The city hall was beautifully decorated, and there the lovely maidens and gallant gentlemen of modern Rome gathered to welcome Georgia's Doctors assembling in council. Dr. T. O. Powell, of Milledgeville, the retiring President, being detained by sickness among his staff, the meeting was called to order by Vice-President G. W. Mulligan, M. D., of Washington.

A short time was allowed for the members present to register and pay their dues. The Secretary gave each one as he came forward a badge consisting of a rosette of white ribbon with old gold centre and a pendant white ribbon bearing the name of the Association and the date of meeting. Each member was also given a free pass over the Rome Street Railroad and dummy line good for three days. These were provided by the local committee, who announced that the members of the Association were welcomed to the open hospitalities of Rome and her citizens.

Dr. Robert Battey, as spokesman for the resident physicians, delivered the address of welcome. The Mayor, W. F. Ayer, then introduced Col. John T. Graves, editor of the *Tribune of Rome*, who extended the hospitalities of the city in a speech replete with wit and sparkling with gems of bright and vivacious humor.

Dr. J. S. Todd, of Atlanta, was called upon to supply the place of Dr. Eugene Foster, of Augusta, and in a most acceptable manner he replied for the Association to the address of welcome.

Dr. R. B. Headden, of Rome, invoked God's blessing on the convention in a short but beautiful and touching prayer.

Vice-President Mulligan then introduced the President-elect, Dr. A. G. Whitehead, of Waynesboro, who read an interesting address, giving the early history of the Association, its trials and struggles, and tracing its triumphant progress to the present time, with mention of the great men who had been members of it. At its conclusion the Association adjourned for dinner.

In the afternoon the regular order of business was begun. The orator of the meeting, J. M. Hull, M. D., of Augusta, was absent n account of sickness.

On motion, the Committee on Expert Testimony was discharged from further service.

The Committee on Medical Legislation was likewise discontinued. The Committee on Prize Essays reported progress and their report was adopted.

Dr. J. T. Galt, of Norfolk, Va., a fraternal delegate from the Virginia Medical Association, made a pleasant talk.

On call of sections reports were received from the first, fifth, seventh, eighth and tenth districts.

The Treasurer's report showed a cash balance of $487.33.

Voluntary papers were read by Dr. J. S. Todd on "Is the Germ Theory of Disease Rational;" by Dr. Virgil O. Hardon on "Superinvolution of the Uterus following Trachelorrhaphy;" by Dr. T. M. Holmes, of Rome, on "Multiple Neuritis," with patient exhibited; by Dr. G. H. Rohé, of Baltimore, on "Electrolysis," illustrated by instruments used; by W. F. Westmoreland, M. D., on "Fissure of the Anus, so called;" by G. H. Noble, M. D., on "A More Rational Method of Treating Flexions of the Uterus;" by Dr. R. C. Word, "The Case of a Medical Friend," asking opinions of members; by Dr. H. P. Cooper on "Hemorrhoids;" by K. P. Moore, M. D., on "Antipyrine;" by Dr. J. B. Medlock, Austell, on "Austell, Ga., as a Health Resort;" by Dr. A. W. Calhoun, of Atlanta, on "Nervous Troubles Dependent Upon a Want of Equilibrium of the Ocular Muscles."

The following papers were read by title: Dr. T. S. Dekle, of Thomasville, "Antipyrine in Gynecological Practice;" Dr. R. O. Cotter, "Some Cases of Hypertrophic Rhinitis with Results of Operation for Same."

Dr. A. C. Davidson, of Sharon, four papers: "Four Cases of Puerperal Eclampsia with Treatment;" "Two Cases of Salivary Calculus, one in Wharton's, the other in Steno's Duct;" "A Case of Puerperal Tenatus after Craniotomy—Death on Eighth day;"

"On Rheumatism, with Cases Illustrating the Value of Oil of Gaultheria in acute inflammatory cases."

By J .McF. Gaston, M. D., of Atlanta, "Laparotomy, with Attachment of Ileum to Incision in a Case of Volvulus."

By Dr. W. F. Westmoreland, Jr., on "Plaster of Paris Splints in the Treatment of Fractures," and "Antiseptic Surgery."

"Scirrhus Cancer of Ascending Colon at Sigmoid Flexure, followed by Rupture of Transverse Colon and Death."

By R. H. Taylor, M. D., "Report of Surgical Cases."

Dr. J. S. Todd read a most eloquent and touching memorial to the late Dr. James A. Gray. It was adopted by a unanimous and rising vote. On motion, it was ordered printed in the Transactions and in THE ATLANTA MEDICAL AND SURGICAL JOURNAL; also, in recognition of the fact that Mrs. Gray was a native of Rome, in the *Rome Tribune.*

The following new members were admitted to the Association:

Drs. J. A. Butts, Brunswick; C. C. Hart, Cross Keys; M. J. Dudley, Sonora; C. F. Griffin, Cassville; Lindsay Johnson, Cartersville; H. Burford, Brunswick; W. X. Bleakney, Eden; G. R. West, Rome; C. A. Brooks, Americus; N. C. Alston, Jr., Richland; L. M. Crichton, Atlanta; D. H. Ramsaur, Floyd county; L. P. Stephens, of Atlanta; D. W. Scott, McDonough; W. O. Dobbins, Fort Gaines; H. P. Quillian, Arp, Ga.; L. J. Belt, Holcomb; H. D. Conyers, Stilesboro; J. A. Dunwody, Brunswick; S. W. Johnson, Graham; J. M. Crawford, Atlanta; T. E. Drewry, Griffin; H. B. McMaster, Waynesboro; J. B. Medlock, Austell.

The most important new business of this meeting was the following resolution of Dr. S. C. Benedict, of Athens, which, after an exciting debate, was adopted without a dissenting voice:

1. *Be it resolved,* That a committee on programme be appointed annually by the Association, consisting of seven members, who shall have power to formulate the programme for the workings of the Association in its meetings, and in so doing shall cooperate with the local committee.

2. That the title to all papers to be read by members of the Association shall be sent to the chairman of the committee at least thirty days before the meeting of the Association, and that this committee appoint and notify the members who shall lead in the discussion of such papers, and shall notify all members of the Association as to the programme to be followed.

3. That this resolution shall not in any way or manner abridge the right of any member to read a voluntary paper or to take part in these discussions.

The committee consists of H. P. Cooper, S. C. Benedict, T. M. Holmes, J. J. Hill, W. H. Doughty, Jr., W. F. Westmoreland, Jr., K. P. Moore.

The committee on re-organization of the Association was discharged.

Rome entertained her visitors well. There were concerts by Shorter College and the Rome Female College, exhibitions of Floyd's products and premium exhibits, and the freedom of the street car and dummy lines. On Thursday afternoon the Association was tendered an old-fashioned Barbecue and Brunswick Stew at Lakeside Park. Returning at 4 p. m., they boarded the steamer Hill City for a delightful excursion down the Coosa river. On Thursday night, from 8 to 11 p. m., the members were tendered receptions by Drs. Robert Battey and J. B. S. Holmes at their respective houses. Everything that could delight and entertain the guests was lavishly displayed, and the entertainments will remain a pleasant memory to all who attended the thirty-ninth annual session.

The following is a list of the incoming officers and committees:

President, J. S. Todd, Atlanta.

First Vice-President, J. B. S. Holmes, Rome.

Second Vice-President, E. R. Anthony, Griffin.

Secretary, K. P. Moore, Macon.

Censor, B. R. Doster, Blakely.

The President announced the following appointments of committees and orator:

Committee on Publication—K. P. Moore, E. C. Goodrich, T. O. Powell, H. McHatton, W. F. Holt, Wm. O'Daniel.

Committee on Necrology—Eugene Foster, G. W. Holmes, H. P. Cooper, R. J. Nunn, M. H. O'Daniel.

Committee on Prize Essays—R. J. Nunn, W. A. Love, H. F. Campbell, Robert Battey.

Committee of Arrangements—W. F. Holt, C. H. Hall, W. R. Winchester, H. J. Williams, H. McHatton.

Orator, T. M. Holmes.

The meeting at Rome was a success. The attendance (over eighty were registered) was good, and the character and talents of the body of a very high grade. Over twenty new members were added. The Association has much to congratulate itself on in its young members, who have pushed rapidly to the front.

The fortieth annual session will convene in Macon on the third Wednesday in April, 1889.

Very truly, etc., H. H.

*Rome, Ga., April 20th, 1888.*

